# International Medical Congress

**Published:** 1886-04-20

**Authors:** 


### INTERNATIONAL MEDICAL CONGRESS.

The editor of the Medical Bulletin remarks, of the feeling abroad in regard to the International Congress, that "The foreign opposition to the Congress, on which the Philadelphia Medical Newas and the New York Medical Record laid so much stress, has vanished like a dream. Three months ago we were informed that 'the way across is long, the fear of the sea is strong,' and that no European physician would think it worth while to cross the Atlantic to attend a 'rump Congress.' We were also informed that all the leading European journals were in opposition to the course of the American Medical Association. The facts of the case are, that the American Medical Association was bitterly attacked by one English journal—the Medical Times and Gazette—but its action so disgusted its subscribers that it has since been compelled to suspend publication. * * *

"Dr. Horatio R. Bigelow, in a recent letter from Vienna to the Journal of the American Medical Association, says: 'The enthusiasm here over the Congress grows daily, and I am quite sure Vienna will send a large delegation to Washington. I hope to be able to establish equal interest in Buda-Pesth and Munich.'

"Letters from London, Dublin, Edinburgh, Paris, and Berlin also assure us that the most prominent physicians of England, Ireland, Scotland, France, and Germany have signified their intention to be present at the meeting of the Congress next year."

